# Dynamic Effects of *Vibrio tubiashii* Infection on Pathology, Transcriptome, and Immunology in the Hepatopancreas of Ivory Shell (*Babylonia areolata*)

**DOI:** 10.3390/biology15130992

**Published:** 2026-06-24

**Authors:** Chen Dai, Dapeng Luo, Qingming Liu, Jing Cui, Yongcai Fu, Haohan Mi, Shihao Yan, Zhongzheng Fu, Guangyuan Xia, Zhigang Tu, Minghui Shen

**Affiliations:** 1 Hainan Provincial Key Laboratory of Tropical Maricultural Technologies, Hainan Academy of Ocean and Fisheries Sciences, Haikou 571126, China; 2 Key Laboratory of Utilization and Conservation for Tropical Marine Bioresources of Ministry of Education, Hainan Tropical Ocean University, Sanya 572022, China; 3 College of Fisheries and Life Sciences, Dalian Ocean University, Dalian 201306, China; 4 School of Marine Biology and Fisheries, Hainan University, Haikou 570228, China; 5 Yazhou Bay Innovation Institute of Hainan Tropical Ocean University, Sanya 572022, China

**Keywords:** *Vibrio tubiashii*, *Babylonia areolata*, transcriptome, hepatopancreas, immune response

## Abstract

*Vibrio tubiashii* infection has led to several *Babylonia areolata* pandemics on the southeast coast of China, yet the immune response of the ivory shell against *V. tubiashii* and the specific pathogen–host interaction remain unclear. This dynamic study aimed to characterize the response of *B. areolata* to *V. tubiashii* infection with the use of pathology, transcriptomics, an enzymatic assay, and inflammatory cytokines. Hepatopancreatic cells showed marked vacuolar degeneration with intact cell membrane and extensive cytoplasmic vacuolization after infection. The dynamic transcriptome of the hepatopancreatic tissue was analyzed by RNA-seq after *V. tubiashii* infection, and a total of 2733 (3 h), 5610 (24 h), 3323 (48 h), and 418 (72 h) differentially expressed genes (DEGs) were identified during infection. The GO and KEGG analyses showed that the DEGs were enriched in metabolic regulation, lysosome, and multiple immune-related pathways such as the MAPK signaling pathway. The immune response of *B. areolata* was distinct, where the early stage of immune response (3 h) showed binding, focal adhesion, and apoptosis, as well as an activated antioxidant system. Here, expression of TNF-α, IL-1, and IL-8 was significantly increased in the hepatopancreas, whereas expression of IL-6 and IL-17 increased afterward. During the middle stage (24 h and 48 h), a large number of DEGs were suppressed, especially those associated with metabolism and lysosomes, although their expression returned to normal during prolonged infection (72 h). The PPI network showed that *ppp2*, *atp6*, and *sos1* were the top immune-related DEGs during infection. Key infection-related and time-course-related genes were analyzed by WGCNA. This study illustrates that oxidative stress, inflammation, and apoptosis are strategies of the hepatopancreatic immune response in *B. areolata* against *V. tubiashii* infection and enlightens conservation and production by furthering our understanding of gastropod immunity.

## Simple Summary

*Vibrio tubiashii* has caused frequent disease outbreaks in *Babylonia areolata* along China’s southeast coast, while the host immune mechanisms and pathogen–host interaction remain poorly understood. Here, we investigated the immune response of *B. areolata* to *V. tubiashii* infection using pathological observation, transcriptomic analysis, enzyme activity detection, and inflammatory cytokine measurement. Post-infection hepatopancreatic cells exhibited obvious vacuolar degeneration with extensive cytoplasmic vacuolization. Transcriptome profiling of hepatopancreas revealed that differentially expressed genes were mainly enriched in metabolic regulation, lysosome, and multiple immune pathways. Enzymatic activity and inflammatory cytokines were induced during early infection and may accelerate the onset of host immune defense. These results provide important insight into the anti-bacterial responses of shellfish and the network of immune-related signaling pathways that are activated during infection.

## 1. Introduction

The ivory shell (*Babylonia areolata*) is a carnivorous gastropod mollusk and one of the most cultured species in the southeastern coastal provinces of China. In recent years, frequent incidents of mass mortality have threatened the development of the *B. areolata* industry, resulting in huge economic losses [1,2,3]. Although almost no obvious clinical signs were present at the onset of the “acute death” of *B. areolata* populations, after 2~3 days, a large number of snails became lethargic on the sand surface, did not drill into the sand, and died shortly afterwards [4]. Epidemiological investigations revealed complicated environmental stressors and pathogens, where bacterial coinfection (*Vibrio* spp.) was one of the most common and severe threats, resulting in multiple tissue disorders and necrosis [4,5,6,7]. *Vibrio* species are ubiquitous common bacteria in marine environments, where *V. tubiashii* is known to cause mortality in *B. areolata* in the south of China [1,2,8,9]. Thus, exploring how *B. areolata* responds to vibriosis is of the essence to mitigate losses in aquaculture.

Previously, the innate immune system, including hemocytes [10,11] and hemolymph [3], was thought to play important roles in the ivory shell’s response to vibriosis infection. Granulocytes exhibit higher phagocytic activity than hyalinocytes. Both hemocyte phagocytosis and respiratory burst activity rise over time and peak at the early stage of infection [11]. During infection with *Vibrio harveyi*, the total hemocyte count in *B. areolata* initially decreased and then gradually increased, while the hemocyte mortality rate decreased and induced a respiratory burst in *B. areolata* [3]. Although some progress has been made on the immune response of ivory shells to *Vibrio* infection, the mechanisms of *B. areolata* resistance to vibriosis are still unclear. In addition to the role of hemocytes in the immune response, researchers have suggested that the hepatopancreas, which is part of the oxidative energy metabolic system, may influence *B. areolata*’s resistance to vibriosis and environmental stimuli. In ivory shells, the hepatopancreas is a multifunctional organ for both metabolic and immune functions [12,13], which means it could be one of the main target tissues during a *Vibrio* infection. Astaxanthin feeding (100 mg/kg) could enhance host immunity by regulating immune-related enzyme activities and gene expression, and by increasing resistance to ammonia stress in the hepatopancreas of *B. areolata*. The general antioxidant capacity (T-AOC) and acid phosphatase (ACP) of the hepatopancreas were significantly increased alongside alkaline phosphatase (AKP), superoxide dismutase (SOD), and catalase (CAT) activity, whilst the malondialdehyde (MDA) content decreased [14]. Similar results were also observed in prebiotic and taurine-supplemented experiments [15,16]. Hepatopancreatic enzyme activities also play a vital role in the resistance of *Babylonia areolata* to environmental stressors such as salinity, pH, and ammonia nitrogen [12,17,18,19]. With prolonged exposure to stress, vacuoles appeared in the hepatopancreas, while cell volume and intercellular space increased. The activities of SOD and CAT decreased significantly under high concentrations of ammonia-induced stress, while MDA levels increased significantly [13]. Furthermore, the CAT, POD, and ACP activity varied in pathogenesis during an outbreak of vibriosis, implying the joint participation of the immune and digestive systems [20].

In recent years, transcriptomics has been widely introduced to study gene expression and molecular pathways related to external stimulation in aquatic invertebrates. The response of ivory shells to ammonia stress was defined as a dynamic process accompanied by energy redistribution. Differentially expressed genes (DEGs) were engaged in maintaining cellular homeostasis, counteracting oxidative stress, and suppressing metabolic activity [13]. Here, *TRAF6*, *GLUT1*, and *CPT1* may serve as key hub genes, supported by varied hepatopancreatic energy consumption in *B. areolata* when adapted to different substrates [21]. Transcriptomic single-nucleotide polymorphism analyses (SNPs) showed that 5866 SNP-unigenes related to 298 KEGG subset pathways were enriched, where “Endocytosis” contained the most SNP-unigenes under LPS stress conditions [22]. Transcriptomic studies have identified several immune-related genes and signaling pathways when challenged by *V. alginolyticus*, *V. parahaemolyticus*, and *V. splendidus*, particularly in *Litopenaeus vannamei* [23,24], *Mytilus galloprovincialis* [25], and *Crassostrea gigas* [26].

The hepatopancreas is the key multifunctional organ in gastropod mollusks, integrating digestive metabolism, nutrition, detoxification, and immune response simultaneously. For *B. areolata*, besides heamolymphocytes, the hepatopancreas is the primary tissue targeted by invading pathogenic *V. tubiashii* and defense bacterial infection [27]. A temporal sampling strategy enables continuous monitoring of dynamic molecular and physiological changes throughout the infection process. Therefore, to systematically investigate *B. areolata* responses against *V. tubiashii* infection, hepatopancreas were collected at 3 h, 24 h, 48 h, and 72 h post bacterial challenge, arranged to cover the early acute response immediately after pathogen invasion, continuous infection stress, and the late infection stage. Additionally, DEGs affected during immune and metabolic stress were identified as candidate genes. Results will guide understanding of the hepatopancreas-related immune responses of *B. areolata*, contribute to research on mollusk immune systems, and advise on novel strategies for vibriosis therapy.

## 2. Materials and Methods

### 2.1. Ethics Statement

*Babylonia areolata* were obtained from the Qukou aquaculture breeding farm in Hainan Province, China. All research was conducted in accordance with the protocols of the Ethics Committee of the Hainan Academy of Ocean and Fisheries Sciences and was approved by the Laboratory Animal Ethics Committee of Hainan Academy of Ocean and Fisheries Sciences (EAEC-HAOFS-No. 2025002).

### 2.2. Animals, Bacterial Infection, and Sampling

Healthy ivory shells were randomly collected from the Qukou breeding farm in Hainan and acclimatized in filtered, aerated seawater tanks (80 × 80 × 60 cm) at a constant temperature of 28.8 ± 1.3 °C, pH of 7.89 ± 0.36, dissolved oxygen above 5 mg/L, and salinity of 26 ± 2 for one week before the experiment. A total of 150 ivory shells (shell length of 2.64 ± 0.27 cm and weight of 3.62 ± 0.22 g) were randomly assigned to five experimental groups (30 snails per group). The groups were labeled as follows: the control or PBS group, and four experimental groups infected with *V. tubiashii* for 3 h, 24 h, 48 h, and 72 h.

The *V. tubiashii* FP17 from our previous study [2] was cultured overnight at 30 °C on Luria Bertani (LB) medium (HB0128, Qingdao Hope Bio-Technology Co., Ltd., Qingdao, China) supplemented with 2% NaCl. The bacterial pellet was centrifuged at 3000 rpm for 10 min, washed with PBS, and resuspended in 1× PBS. The infection dose of *V. tubiashii* was screened according to previous results [2,27]. The ivory shells of the experimental *V. tubiashii* groups were each intramuscularly injected with 100 μL of *V. tubiashii* FP17 suspension at a final concentration of 1.2 × 10^7^ CFU/mL. Animals in the control group were injected with an equal volume of PBS. The ivory shells were fasted for 24 h before and after the trial. Three hepatopancreas samples from each group were fixed in 4% formaldehyde at room temperature at the start of the experiment, and the remainder of the ivory shells in each group were sampled at 3 h, 24 h, 48 h, and 72 h after *V. tubiashii* FP17 injection. Specifically, nine snails from each group were randomly selected for hepatopancreatic tissue collection and RNA extraction. Any three of the nine individuals were pooled into one replicate to produce three independent biological replicates, which were independently processed and sequenced, as well as for subsequent qRT-PCR verification. The same sampling protocol was used for the enzyme activity and ELISA tests. All hepatopancreas samples were snap frozen in liquid nitrogen after collection and stored at −80°C.

### 2.3. Histopathology

Hepatopancreatic tissue of *B. areolata* was fixed with 4% formaldehyde for more than 24 h and then dehydrated in incremental dilutions of alcohol and wax leaching. The wax-soaked tissues were embedded in paraffin and cut into 4 μm thick slices, stained with hematoxylin-eosin, and viewed with an inverted phase contrast microscope (Olympus CKX53, Tokyo, Japan).

### 2.4. RNA Extraction and Illumina HiSeq Sequencing

Total RNA was extracted from hepatopancreatic samples using TRIzol^®^ Reagent following the manufacturer’s instructions (Invitrogen, Carlsbad, CA, USA). The RNA quality and concentration were determined using a 2100 Bioanalyzer (Agilent Technologies, Santa Clara, CA, USA), agarose gel electrophoresis, and a NanoDrop (Thermo Fisher Scientific, Wilmington, DE, USA). High-quality RNA was used for library preparation. Sequencing libraries were constructed using the Hieff NGS^®^ Ultima Dual-Mode mRNA Library Prep Kit (Yeasen, Shanghai Yeasen BioTechnologies Co., Ltd., Shanghai, China). Libraries were generated using the Illumina Novaseq6000 platform by Gene Denovo Biotechnology Co. (Guangzhou, China).

### 2.5. Bioinformatic Analysis

High-quality clean reads were obtained from raw reads filtered in fastp (version 0.18.0) [28]. Paired-end clean reads were mapped to the reference *B. areolata* genome (submitted GenBank assembly: GCA_041734735.1) using HISAT2 2.1.0 [29], and the mapped reads of each sample were assembled using StringTie v1.3.1. For each transcription region, a FPKM (fragment per kilobase of transcript per million mapped reads) value was calculated to quantify its expression abundance and variation using RSEM software (v1.3.3).

Differential expression analysis between the two different groups was performed in DESeq2 [30]. Multiple testing correction was carried out according to the Benjamini–Hochberg method. The genes/transcripts with a parameter of false discovery rate (FDR) below 0.05 and an absolute fold change ≥ 2 were considered DEGs. To understand the functions of DEGs, Gene Ontology (GO) and Kyoto Encyclopedia of Genes and Genomes (KEGG) enrichment analysis were carried out. The DEGs were considered significantly enriched in GO terms and pathways depending on their Benjamini–Hochberg corrected *p*-value < 0.05.

To examine the expression pattern of the DEGs, the expression data of each sample (in the order of treatment) were normalized to 0, log2 (v1/v0), log2 (v2/v0), and then clustered in the Short Time-series Expression Miner (STEM) v1.3.13 software program [31]. The parameters were set as follows: (1) the maximum unit change in model profiles between time points was 1; (2) maximum output profiles was 20 (similar profiles will be merged); and (3) minimum ratio of fold change of DEGs was no less than 2.0. The clustered profiles with a *p*-value ≤ 0.05 were considered significant profiles. Then, the DEGs in all or each profile were subjected to Gene Ontology (GO) and KEGG pathway enrichment analysis. Through *p*-value calculation and FDR correction, the GO terms or pathways with a Q-value ≤ 0.05 were defined as significantly enriched.

To further analyze the inter-cooperative relationship of the DEGs’ encoded proteins, the STRING [32] database (offline version deployed on the commercial sequencing cloud platform) was used to construct the protein–protein interaction (PPI) networks. Because *B. areolata* is a non-model species, protein interactions were inferred by homology using the available reference species *Pomacea canaliculata* as the background. Interactions satisfying combined score ≥ 300 were retained. The combined score represents the integrated confidence score ranging from 0 to 1000. Node degree calculation and gene ranking were performed on this final interaction set.

### 2.6. Weighted Gene Correlation Network Analysis (WGCNA)

WGCNA [33] was employed to construct a weighted gene co-expression network comprising all genes. The expression matrix of all genes was standardized by log2 (FPKM1), and the median absolute deviation (MAD) of each gene was calculated. The appropriate weighting coefficient β = 4 was calculated as the power value to satisfy the scale-free topology of the network. Modules were constructed with a minimum module size of 50, and modules with highly correlated eigengenes were merged using the default cut height of 0.25. The module eigengene (ME) of each module was then correlated with the measured phenotypic traits, and the correlation coefficients and corresponding *p*-values were visualized in a heatmap.

### 2.7. Validation of DEGs by qRT-PCR

To validate the Illumina sequencing data, the expression level of 15 DEGs was assessed using qRT-PCR on the CFX96TM real-time system C1000 touch thermal cycler (Bio-Rad, Hercules, CA, USA). The genes were selected mainly according to expression fold changes during *V. tubiashii* infection, and immune-related function was also considered. Beta-actin (β-actin) was used as an internal reference gene, and the primers are listed in Table 2. The RNA for RNA-seq was synthesized into cDNA using the HiScript III RT SuperMix for qRT-PCR (R323, Vazyme Biotech Col, Ltd., Nanjing, China). Each 20 μL reaction mixture consisted of 10 μL 2 × ChamQ Universal SYBR qPCR Master Mix, 0.4 μL of each primer (10 μM), 2 μL cDNA, and 7.2 μL ddH_2_O (Q712, Vazyme Biotech Col, Ltd., Nanjing, China). The PCR cycling parameters included an initial denaturation step at 95 °C for 30 s, followed by 40 cycles of denaturation at 95 °C for 10 s and annealing at 58 °C for 30 s. The relative expression levels of the DEGs were calculated using the 2−ΔΔCT method. All PCR reactions were conducted in triplicate.

### 2.8. Enzymatic Assays

The hepatopancreas samples were weighed and homogenized in precooled 0.9% saline according to the manufacturer’s instructions. The homogenates were centrifuged at 3000 rpm at 4 °C for 10 min, and the supernatant was collected for the enzymatic assays. The hepatopancreas superoxide dismutase (SOD) (A001–3), catalase (CAT) (A007-1-1), acid phosphatase (ACP), alkaline phosphatase (AKP) (A059–2), lysozyme (LZM) (A050-1-1), peroxidase (POD) (A084-1-1), glutathione peroxidase (GSH-PX) (A005-1), lipase (LPS) (A054-2-1), pepsin (A080-1-1), amylase (AMS) (C016-1-1), Na^+^K^+^ATPase (A070-2), and malondialdehyde (MDA) (A003-1) contents were measured following each kit’s instructions (Nanjing Jiancheng Bioengineering Institute, Nanjing, China). The protein concentration of each sample was determined using the Coomassie brilliant blue Protein Assay Kit (A045-2).

### 2.9. ELISA Test

The hepatopancreas samples were weighed and homogenized in 0.9% saline and centrifuged at 3000 rpm at 4 °C for 10 min. The supernatant was collected for the ELISA test. Shellfish interleukin 1 (IL-1) (MM-927944O1), IL-6 (MM-1040X1), IL-8 (MM-1046X1), IL-10 (MM-1050X1), and tumor necrosis factor alpha (TNF-α) (MM-928667O1) were measured following each kit’s instructions (Jiangsu Meimian Industrial Co., Ltd., Nanjing, China) using a microplate reader F50 (Tecan Sunrise Microplate Reader, Männedorf, Switzerland).

### 2.10. Statistical Analysis

Results were expressed as the mean ± standard error of the mean. For enzyme activity and cytokine assays, the normality of residuals was primarily assessed using the Shapiro–Wilk test. The homogeneity of variances was verified using the Brown–Forsythe test. For datasets meeting the assumptions of normality (Shapiro–Wilk *p* > 0.05) and equal variance (Brown–Forsythe *p* > 0.05), ordinary one-way ANOVA was performed, followed by Dunnett’s multiple comparison test to compare with the PBS control. Differences of *p* < 0.05, *p* < 0.01, or *p* < 0.001 were considered significant. All statistical analyses were performed in GraphPad PRISM 9.0 (San Diego, CA, USA).

## 3. Results

### 3.1. Histopathology of B. areolata Hepatopancreas During V. tubiashii Infection

**Figure 1:**
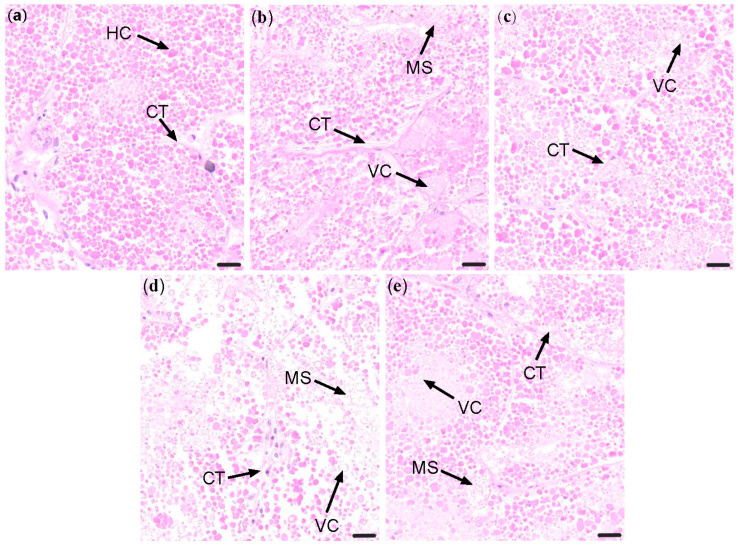
Histopathology of the *B. areolata* hepatopancreas in control group (**a**) and *V. tubiashii* infected groups at 3 h (**b**), 24 h (**c**), 48 h (**d**), and 72 h (**e**). Figures are 400× field of views with 20 μm scale bar. HC: hepatopancreatic cell, CT: connective tissue, MS: meshed structure, VC: vacuolated cells.

The hepatopancreas embeds multiple types of digestive gland tissues. To precisely observe the histopathological alterations of hepatopancreatic cells, the anterior portion of the hepatopancreas was selected for the preparation of pathological sections in this study. The hepatopancreas parenchyma of *B. areolata* consists of many round hepatic lobule-like structures that are separated by connective tissue (Figure 1a). In the control group, the boundaries of the lobules were distinct and compact with full red staining of the anucleate hepatopancreatic cells. In the infected group, widened gaps were visible between connective tissue and hepatopancreatic cells; the hepatic units retained clear outlines and exhibited a mesh-like arrangement. Hepatopancreatic cells displayed lighter staining, alongside local polygonal cell fusion or cellular atrophy. Vacuolar degeneration was observed in hepatopancreatic cells, with intact cell membranes and extensive cytoplasmic vacuolization detected (Figure 1b–e).

### 3.2. RNA-Seq Results, Quality Control, and qRT-PCR Validation

A total of 15 libraries (PBS, 3 h, 24 h, 48 h, and 72 h) were constructed to study the transcriptional response to *V. tubiashii* infection. Qualified clean reads were filtered by removing adaptor sequences, low-quality reads, and reads with N ratio > 10%. The Q30 values of the clean reads were greater than 93% in all libraries, indicating high data quality. The assembled *B. areolata* genome was used as a reference database for mapping the clean reads (submitted GenBank assembly: GCA_041734735.1). After quality control and the removal of mapped rRNA in Bowtie 2, unmapped reads were retained for further analysis. The total number of reads mapped to the reference genome reached a mapping rate of 77% to 83% (Table 1).

**Figure 2:**
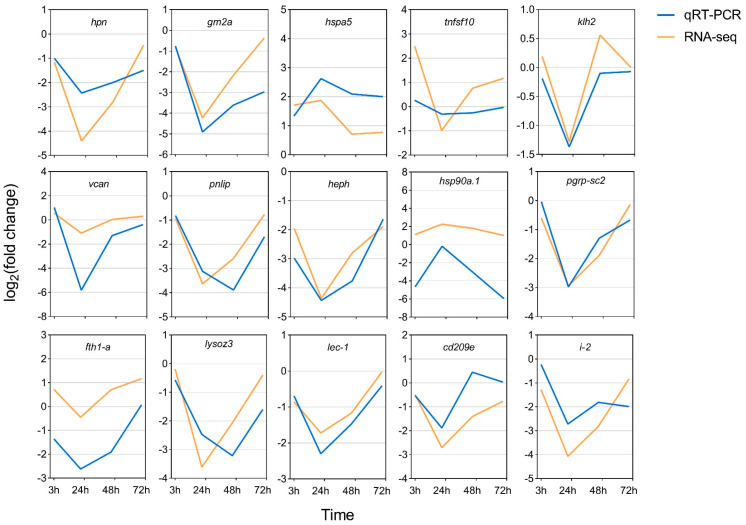
Comparison of relative expression levels determined by RNA-seq and qRT-PCR. β-actin was used as internal control.

To verify the accuracy of the RNA-seq data, 15 genes were selected for validation by quantitative PCR. The selected genes and primers are listed in Table 2. The expression pattern of the tested DEGs was consistent with the RNA-seq data and showed the same pattern (Figure 2).

### 3.3. Differential Expression Analysis

**Figure 3:**
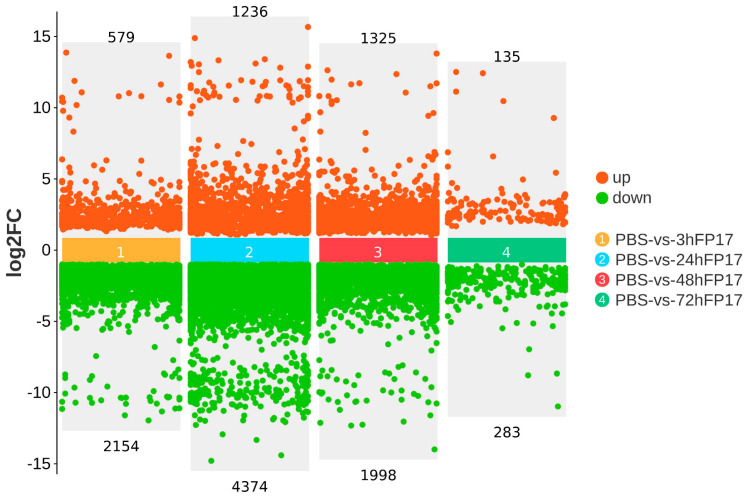
Different expression genes (DEGs) at 3 h, 24 h, 48 h, and 72 h. Groups compared with PBS control group in hepatopancreas of *B. areolata*.

**Figure 4:**
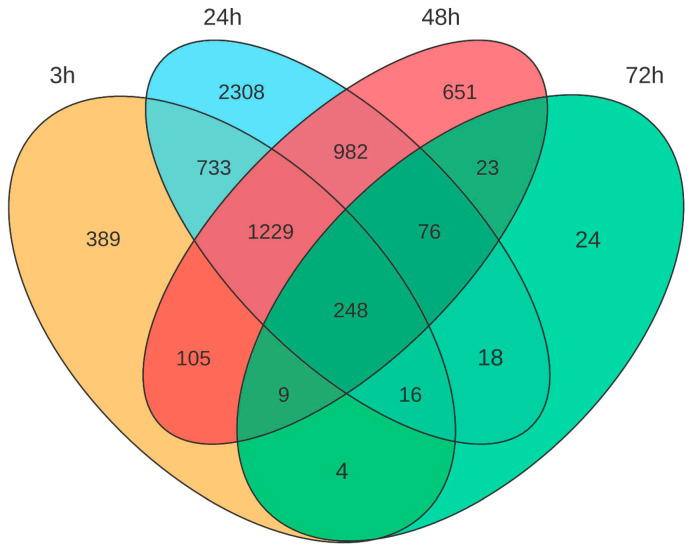
The Venn diagram of the DEGs at 3 h, 24 h, 48 h, and 72 h infected groups.

To define the dynamic ivory shell transcriptome profile after *V. tubiashii* FP17 infection, DEGs were detected at 3 h, 24 h, 48 h, and 72 h post infection and compared with the PBS control. A total of 2733 (3 h), 5610 (24 h), 3323 (48 h), and 418 (72 h) DEGs were identified, respectively. Among these, 579 DEGs were up-regulated and 2154 DEGs were down-regulated at 3 h after infection; 1236 DEGs were up-regulated and 4374 DEGs were down-regulated at 24 h after infection; 1325 were up-regulated and 1998 were down-regulated at 48 h after infection; and 135 were up-regulated and 283 were down-regulated at 72 h after infection (Figure 3). The abundant DEGs detected within 48 h correspond to the robust activation of acute innate immunity and stress response triggered by pathogen invasion. By 72 h post infection, the sharp decline in DEG number indicates that the acute immune activation is attenuated and tends to stabilize. This shift represents a transition from intense acute defensive response to steady-state adaptation in molluscan innate immunity. As shown in the Venn diagram, a total of 6815 DEGs from four time points in the experimental groups were selected for further analysis (Figure 4). Among these, 248 DEGs were expressed at all four different time points, most of which were down-regulated except *asph*, *ilcr-2*, and *lrp4*. These DEGs are related to immune system function and involve several receptors such as *tnsf10/11*, *tgfbr1*, and *igf1r*; proteoglycans: *vcan* and *acan*; metabolic factors: *ada2a*, *gnpat*, *smpd1*, and several cytochrome P450 family members; and others: *nfat5*, *trim2*, *map3k2*, *npas4*, and *trp53inp1*.

**Figure 5:**
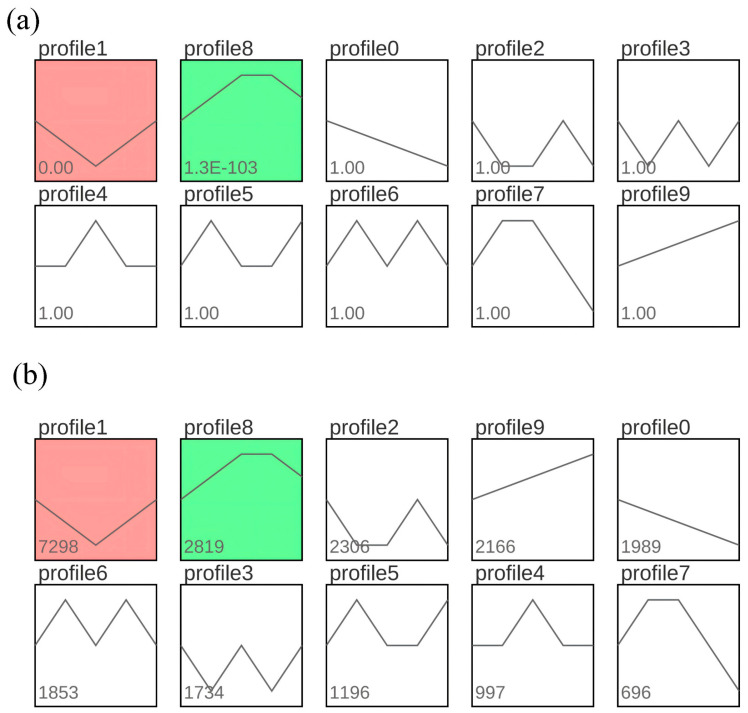
Trend analysis of the DEGs expression trend changes. (**a**) Trend analysis results sorted by *p*-value. (**b**) Trend analysis results sorted by the numbers of DEGs. Boxes filled with red or green denote statistically significantly enriched gene profiles (*p* < 0.05).

Next, 6815 DEGs were analyzed for expression trends using STEM. There were 10 different expression trends, with two significantly enriched profiles: profiles 1 and 8 (Figure 5). These two showed opposing expression trends, where profile 1 contained the most genes (3667) and varied most significantly, displaying an initial downregulation (3 h and 24 h) and then upregulation (48 h and 72 h), while profile 8 contained 1293 DEGs and was up-regulated initially and then down-regulated. These may represent the early and late immune defense waves. Profile 8 was analyzed further since the DEGs in this profile may be involved in early host defense. The significantly up-regulated genes *sytl5*, *xiap*, and *tgm1* peaked at 3 h, while *cryab*, *hpn*, *slc43a3*, *esco2*, *pgm1*, *cdca3*, *diap1*, *arf4*, *aoc3*, and *cav1* peaked at 24 h. The most significant DEGs at 48 h were *gpx6*, *nxn*, *adam10*, *ywhab*, *ten-m*, *pgbd2*, *slc66a3*, and *stk38*. Notably, *xiap*, *diap1*, *cryab*, and *ywhab* can inhibit apoptosis and regulate inflammation and immune cell survival. Moreover, *gpx6*, *nxn*, *slc66a3*, and *stk38* are associated with antioxidant function that may protect cells from oxidative damage. The solute carrier (SLC) family members *slc43a3* and *slc66a3* were also up-regulated and may participate in cellular antioxidant defense and enhanced cell viability. Unexpectedly, most metabolic and energy-related genes were down-regulated in profile 1, including many genes associated with glycolysis and fatty acid oxidation, such as *cpt1*, a specific transporter required for the entry of fatty acids into the mitochondria for β-oxidation.

### 3.4. GO Enrichment of DEGs

**Figure 6:**
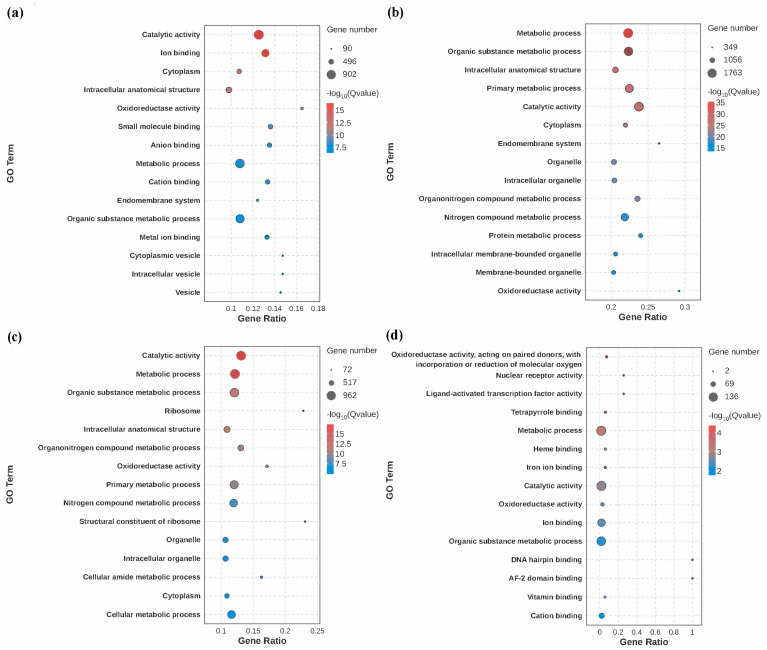
GO enriched circle plot of the top 15 pathways at 3 h vs PBS (**a**), 24 h vs PBS (**b**), 48 h vs PBS (**c**), and 72 h vs. PBS (**d**) after *V. tubiashii* infection.

To identify the potential function of these DEGs, GO and KEGG enrichment analysis were performed on the clustered DEGs. A total of 6815 DEGs were grouped into Biological Process (BP), Cellular Component (CC), and Molecular Function (MF) (Figure 6). The top terms of the three categories were identified in binding, cellular process, metabolic process, catalytic activity, localization, and response to stimulus, which may relate to the bacterial infection. At the beginning of infection (3 h), the DEGs were significantly enriched in catalytic activity, ion binding, oxidoreductase activity, and intracellular anatomical structure besides cytoplasm and metabolic process (Figure 6a). At 24 h after infection, various metabolic processes were the most enriched, followed by catalytic activity, cytoplasm, organelle, proteolysis, and organelle and vesicle-mediated transport (Figure 6b). In the long-term (48 h and 72 h), lysosome, ribosome, and lytic vacuole were highly enriched. Signaling, transporter activity, and immune system processes were also highly enriched (Figure 6c,d).

The up- and down-regulated DEGs on the annotated GO were further analyzed and revealed distinct expression patterns among the groups. A large number of genes were down-regulated at 3 h, 24 h, and 48 h in most of the GO terms, including several types of binding, oxidoreductase activity, and the endomembrane system. Significantly up-regulated DEGs at 3 h included DNA polymerase and nucleotidyltransferase activity, mechanosensitive ion channel activity, and peptidase activity. In contrast, at 24 h and 48 h, DEGs were significantly up-regulated in peptide biosynthetic process, amide biosynthetic process, ribosome, and translation. Only a few genes were highly expressed at 72 h, while those in oxidoreductase activity, binding, and nuclear receptor activity were down-regulated.

### 3.5. KEGG Enrichment of DEGs

**Figure 7:**
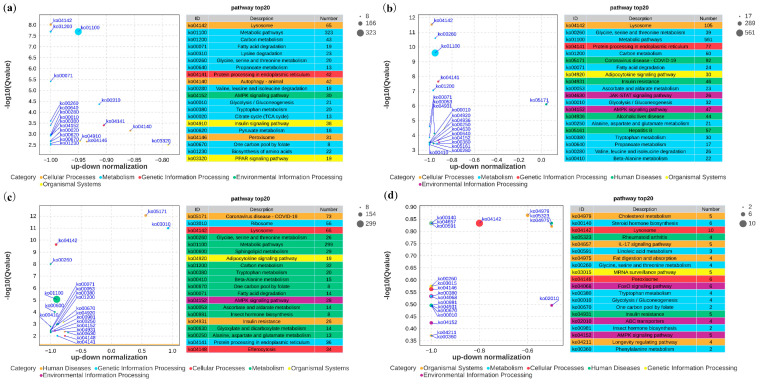
KEGG enriched bubble chart of the top 20 pathways at 3 h (**a**), 24 h (**b**), 48 h (**c**), and 72 h (**d**) after infection.

The top 20 enriched pathways are shown in Figure 7. Generally, a large number of genes were significantly down-regulated compared to up-regulated during infection, where the most enriched were lysosome and metabolic pathway. The up-regulated genes in the 3 h group were significantly enriched in lipid metabolism, NOD-like receptor signaling pathway, apoptosis, and focal adhension, while down-regulated genes were enriched in fatty acid degradation, autophagy, peroxisome, insulin signaling pathway, and adipocytokine signaling pathway, which were also enriched in the 24 h group (Figure 7a,b). The protein processing in endoplasmic reticulum, metabolism, including amino acid, carbon, fatty acid, and insulin pathways, was also enriched at 24 h (Figure 7b). Moreover, ribosome-related pathways showed the most prominent enrichment among upregulated genes at 24 h and 48 h, indicating that enhanced protein synthesis and signal transduction act as key responses during the middle stage of infection (Figure 7b,c). However, up-regulated genes were significantly enriched in phagosome and apoptosis in the 24 h group, while neutrophil extracellular trap formation, necroptosis, and toll-like receptor signaling pathway were enriched in the 48 h group, highlighting the distinct functions of the immune defense system after infection (Figure 7b,c). In contrast, at 72 h, down-regulated genes were significantly enriched in cholesterol metabolism, steroid hormone biosynthesis, and immune-related pathway (Figure 7d). Up-regulated genes were enriched in phagosome, fat digestion, and absorption. The pathways annotated as coronavirus disease in the database cover multiple functional modules conserved in invertebrates, including TNF signaling, FcγR-mediated phagocytosis, toll-like receptor signaling, as well as complement and coagulation cascades process. The other enriched KEGG pathways related to host metabolism and response to infection were also identified, including the AMPK signaling pathway, MyD88-dependent pathway, FoxO signaling, and PI3K-Akt signaling. The top 20 pathways related to immunity at different time points are summarized in Table 3. Notably, several vertebrate immune pathways and human-disease-associated pathways appeared in the top 20 enriched KEGG pathways (Table 3). The enrichment of vertebrate-specific pathways is an annotation bias inherent to KEGG, which conducts mapping based on sequence homology and is dominated by vertebrate reference data, rather than authentic functional pathways in mollusks.

### 3.6. Network Construction and Screening of Key Genes

**Figure 8:**
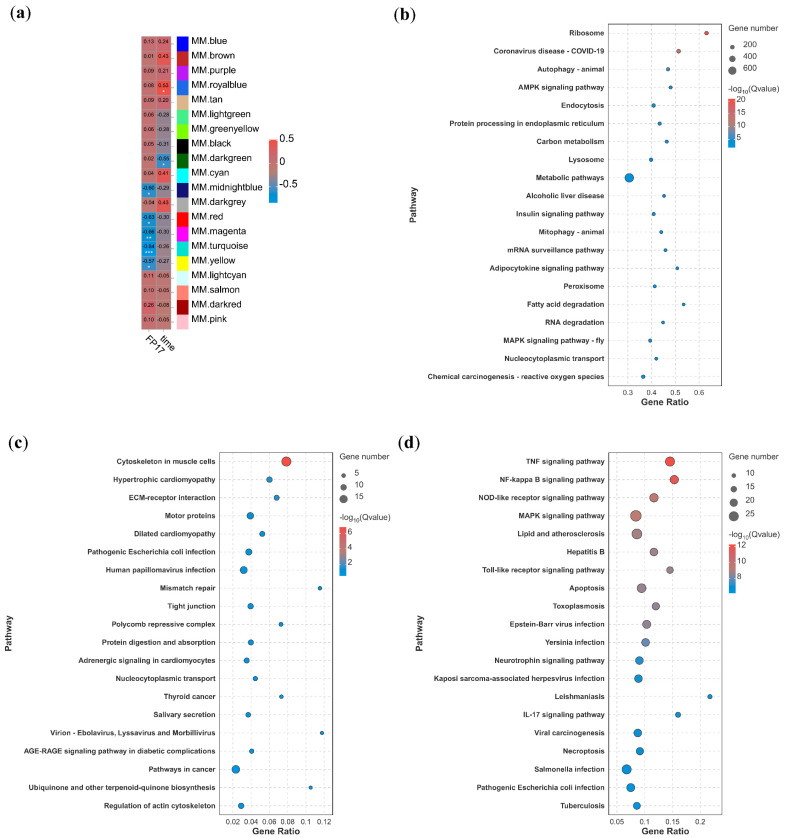
Weighted gene co-expression network analysis (WGCNA) of *V. tubiashii* infection in a time-course. (**a**) Heatmap of correlation coefficient in different modules that related to FP17 infection and duration. Asterisks within cells denote statistical significance of correlation: * *p* < 0.05, ** *p* < 0.01, *** *p* < 0.001. KEGG enrichment pathway of turquoise (**b**), royalblue (**c**), and darkgreen (**d**) modules.

To elucidate specific co-expression modules associated with the progression of *V. tubiashii* infection, all the identified transcriptomes were analyzed by WGCNA (Figure 8a). The turquoise module showed the most significant negative correlation (r = −0.84) with *V. tubiashii* infection, where KEGG terms were enriched in ribosome, autophagy, AMPK signaling pathway, and endocytosis (Figure 8b). The royal blue module was the most significantly positively correlated module (r = 0.53), whereas the dark green module was the most significantly negatively correlated module (r = −0.55) with infection time. Here, cytoskeleton in muscle cells and ECM-receptor interaction were enriched and positively correlated, while TNF signaling pathway, NF-kappa B signaling pathway, and NOD-like receptor signaling pathway were enriched and negatively correlated with infection progression (Figure 8c,d). The top 1% of genes in the turquoise, royal blue, and dark green modules were considered key genes and were highly correlated with *V. tubiashii* infection (Table 4).

### 3.7. Construction of Immune-Related Protein Interaction Networks

In addition to WGCNA, a protein–protein interaction analysis was performed to identify proteins critical to the immune response. The 259 DEGs were selected from the significantly enriched immune-related KEGG pathways for the PPI network construction, and the top-ranked genes that were sorted by node degree value and immune-related role are summarized in Table 5. Protein phosphatase 2 (*ppp2* encoded) shows the highest degree, followed by ATP synthase (*atp6* encoded) and guanine nucleotide exchange factor (*sos1* encoded). Meanwhile, *drk* (homolog to *grb2*), *grb2*, *atf2*, *jun*, and *polr3* with higher degree were also confirmed. The above genes are reported functions in multiple biological processes linked to molluscan defense, including transcriptional regulation and stress response, energy metabolism and mitochondrial function, protein phosphorylation and signal regulation, RNA synthesis, as well as the Ras-MAPK signaling pathway. Therefore, these genes may serve as candidate genes for further functional characterization in future research.

### 3.8. Changes in Enzyme Activity in the B. areolata Hepatopancreas

**Figure 9:**
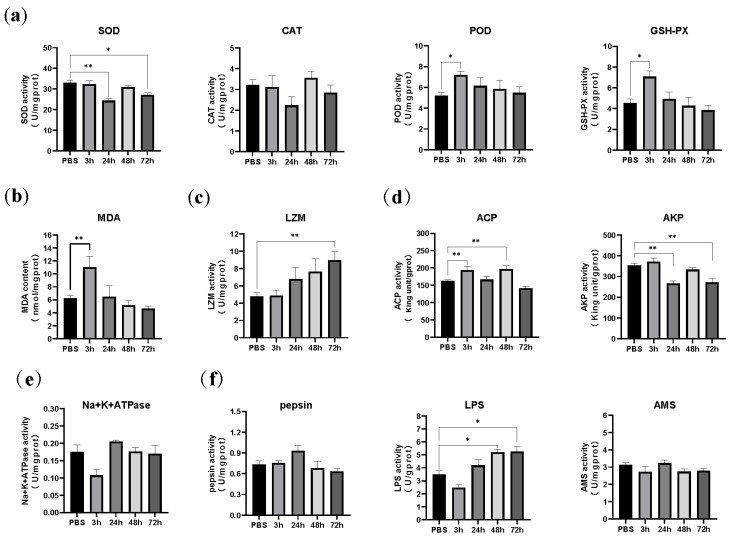
The time-course alteration of different enzyme activity in hepatopancreas upon *V. tubiashii* infection. (**a**) Oxidative-stress-related enzyme (SOD, CAT, POD, and GSH-PX) activity; (**b**) malondialdehyde (MDA); (**c**) lysozyme (LZM); (**d**) acid phosphatase (ACP) and alkaline phosphatase (AKP); (**e**) Na^+^/K^+^-ATPase; (**f**) pepsin, lipase (LPS), and amylase (AMS). Data are presented as mean ± standard error of the mean (SEM). Each group has *n* ≥ 3 independent biological replicates. Ordinary one-way ANOVA combined with Dunnett’s multiple comparisons test was used for statistical analysis. * *p* < 0.05 and ** *p* < 0.01 indicate significant differences compared with the PBS control group.

To visualize the effect of *V. tubiashii* infection on the snail’s antioxidant defense system, the SOD, CAT, POD, GSH-PX activities, and MDA content were tested (Figure 9a,b). The dynamics of SOD and CAT activity were quite similar as infection progressed. Both decreased initially, reaching their lowest levels at 24 h (24.340 ± 0.964 U/mgprot and 2.231 ± 0.420 U/mgprot, respectively) but returned to normal levels by 48 h and then decreased again at 72 h. At 24 h of infection, bacteria or toxic substances multiply and accumulate, resulting in a sustained release of ROS, which consumes large amounts of SOD and CAT. On the other hand, the increased cell damage might also inhibit enzyme synthesis, at which point antioxidant capacity was at its lowest.

Conversely, POD, GSH-PX, and MDA increased significantly at 3 h and gradually decreased to normal (Figure 9a,b). The enzymatic activities of POD and GSH-PX peaked at 7.200 ± 0.344 U/mgprot and 7.083 ± 0.549 U/mgprot, respectively. The results indicated that POD and GSH-PX, prior to SOD and CAT, remove ROS in the hepatopancreas of *B. areolata* during the *V. tubishii* infection. MDA content peaked (11.050 ± 1.679 U/mgprot) at 3 h, which echoes the early elevation of POD and GSH-PX, suggesting that ROS has triggered lipid oxidation in the cell membrane in the early stages of infection, and the elevation of antioxidant enzymes may be a compensatory response to this injury. Additionally, the activity of immune-defense-related enzyme LZM increased gradually with infection, reaching its highest value of 8.980 ± 0.970 U/mgprot at 72 h (Figure 9c). In the hepatopancreas, ACP increased initially, decreased by 24 h, and then increased again and subsequently recovered. The activity of ACP increased significantly at 3 h and 48 h to 193.80 ± 10.05 King unit/gprot and 197.20 ± 9.808 King unit/gprot, respectively (Figure 9d). On the contrary, AKP activity displayed a similar trend to SOD and CAT, reaching its lowest level at 24 h (266.00 ± 12.73 King unit/gprot) and 72 h (271.50 ± 17.03 King unit/gprot) (Figure 9d). The sensitive indicators of membrane function, Na^+^K^+^-ATPase, whose activity had only slightly decreased by 3 h post infection, were tested further (Figure 9e). Results showed that, in combination with previous MDA changes, lipid peroxidation could directly damage the Na^+^K^+^ATPase protein structure on the cell membrane.

Furthermore, as the hepatopancreas is a digestive gland, the activities of pepsin, lipase, and amylase were also tested on *V. tubiashii* infection (Figure 9f). Lipase activity decreased at 3 h but subsequently increased, possibly due to the metabolic reprogramming of energy metabolism in infection states driven by energetic demand. Unlike lipase, the activities of pepsin and amylase did not change much and were only slightly increased at 24 h, which may be because of the increased immune demand for proteins (pepsin) and carbohydrates (amylase).

### 3.9. Changes in Inflammatory Factors in the Hepatopancreas

**Figure 10:**
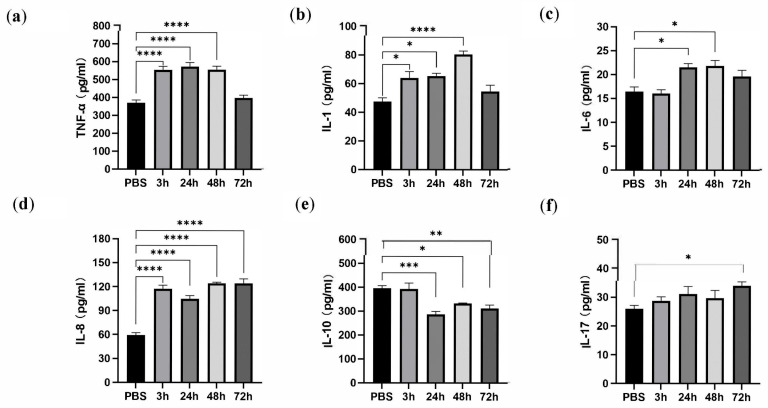
Inflammatory cytokine contents in hepatopancreas were measured by ELISA in a time course after *V. tubiashii* infection. Concentration of TNF-α (**a**), IL-1 (**b**), IL-6 (**c**), IL-8 (**d**), IL-10 (**e**), and IL-17 (**f**), respectively. Data are presented as mean ± standard error of the mean (SEM). Each group has *n* ≥ 3 independent biological replicates. Ordinary one-way ANOVA combined with Dunnett’s multiple comparisons test was used for statistical analysis. * *p* < 0.05, ** *p* < 0.01, *** *p* < 0.001, **** *p* ≤ 0.0001. indicate significant differences compared with the PBS control group.

To define the variation in the inflammatory process, an ELISA was used to test the levels of different inflammation-related cytokines in the *B. areolata* hepatopancreas along the progression of the *V. tubiashii* infection (Figure 10). Similar to vertebrates, TNF-α and IL-1 were the fastest responding cytokines, increasing rapidly to a level of 555.30 ± 18.76 pg/mL and 64.06 ± 4.43 pg/mL, respectively, at 3 h (Figure 10a,b). Since the expression of IL-6 is induced by TNF-α and IL-1, its expression remained unchanged at 3 h and increased significantly to a peak of 21.53 ± 0.79 pg/mL at 24 h and 21.53 ± 0.79 pg/mL at 48 h (Figure 10a,b). With continuous stimulation through infection, TNF-α levels remained high, and IL-1 peaked a second time from 24 h to 48 h. At 72 h, TNF-α and IL-1 levels were significantly decreased, approaching normal, while IL-6 levels descended slowly, probably due to the participation of the tissue repair process (Figure 10c). Surprisingly, IL-8 continued to increase and peaked from 3 h (117.50 ± 4.32 pg/mL) to 72 h (124.40 ± 5.52 pg/mL) (Figure 10d). A persistent increase in IL-8, accompanied by a rapid decline in TNF-α and IL-1 at 72 h, could indicate a recovery phase in inflammation. On the contrary, IL-10 decreased significantly to its lowest level (approximately 286.60 ± 13.24 pg/mL) at 3 h and remained low until 72 h, while IL-17 was only elevated at 72 h, indicating it may be involved in tissue repair rather than inflammation (Figure 10e,f).

## 4. Discussion

*Babylonia areolata*, a major economic shellfish species, has an open circulatory system and a unique immune system, where the hepatopancreas acts as its digestive, metabolic, and immune organ [34]. In this study, DEGs of the *B. areolata* hepatopancreas transcriptome were identified during *V. tubiashii* stimulation. Critical time points post bacterial infection (3 h, 24 h, 48 h, and 72 h) were selected to elucidate the early, medium, and late host responses. The transcriptomic profile revealed a distinct and complex immune response strongly influenced by the progression of the *V. tubiashii* infection following a series of pathophysiological changes, including inflammation, cell damage, oxidative stress, and metabolic disorders.

At the early stage of *V. tubiashii* infection (3 h), transcriptome analysis revealed significant enrichment of NOD-like receptor signaling, apoptosis, and focal adhesion pathways, which are core modules of molluscan innate immunity. Consistent with the transcriptomic activation of immune cascades, pro-inflammatory cytokines were markedly upregulated at this time point. The synchronized elevation of inflammatory mediators and enrichment of immune signaling pathways collectively demonstrate that the host rapidly initiated acute immune and inflammatory responses upon pathogen invasion. The antioxidant system was rapidly and continuously involved. Meanwhile, the altered expression of metabolism-related pathways reflected immediate energy redistribution to support frontline defense. During the middle infection phase (24 h and 48 h), phagosome, Toll-like receptor signaling, necroptosis, and other defense pathways remained highly enriched in the transcriptome. Correspondingly, pro-inflammatory cytokines maintained high levels, indicating continuous activation of antibacterial defense and inflammatory reactions. Alongside persistent immune activation, the host suffered oxidative stress. Transcriptomic changes in peroxisome and fatty acid metabolism pathways reflect disrupted antioxidant balance. Histopathological observations further confirmed gradual aggravation of hepatopancreatic damage during this period. The decreased AKP activity, as a marker of hepatopancreatic function, was associated with ongoing tissue lesions. A sharp reduction in the total number of DEGs was observed at 72 h, indicating that large-scale transcriptional genes gradually tend to stabilize. Combined with the activities of enzymes, the cytokine pattern and the stabilized transcriptome collectively illustrate the status of adaptive regulation and incomplete tissue recovery in the late infection stage. The dynamic changes form a coherent chain from transcriptional alteration to physiological and morphological injury.

Typically, oxidative stress is the main reported response in ivory shells either to *V. harveyi* infection [35] or environmental stressors such as pH and ammonia [12,18]. Similar to previous research, the expression of CAT was significantly decreased with time at the transcriptome level (SOD1 only at 24 h), although *V. harveyi* induced an excessive production of ROS that led to the accumulation of MDA in the hepatopancreas over time [34]. The MDA, POD, and GSH-PX levels were only significantly elevated during the very early stages of infection (3 h) and returned to normal thereafter. Interestingly, even though LZM, ACP, and AKP are all involved in eliminating pathogens, they showed distinct activity patterns, including elevated activity of ACP at 3 h and 48 h and reduced activity of AKP at 24 and 72 h, while the activity of LZM continued to increase over time. This indicates that various enzymes perform correlative functions at different time points in the host’s immune response.

To our knowledge, this study is the first documentation of the levels of inflammatory cytokines in *B. areolata* during infection. We observed marked alterations in cytokine relative abundance and antioxidant enzyme activities at successive infection time points. At 3 h, elevated TNF-α coincided with signatures of oxidative stress. The concurrent sharp increases in POD and GSH-PX may facilitate ROS scavenging, while elevated LZM activity, together with increased IL-1 and IL-8 levels, potentially participates in antibacterial defense. The higher MDA value at this time point indicates lipid peroxidation occurred in hepatopancreatic cell membranes. At 24 h, simultaneous elevation of TNF-α and IL-6 coincided with aggravated oxidative damage and reduced SOD and CAT activity. At 48 h, inflammatory cytokines may trigger limited antioxidant compensation, where sustained damage leads to a renewed decline in enzyme activity at 72 h. The anti-inflammatory role of IL-10 has not been confirmed in shellfish. The observation of unaltered IL-10 levels may suggest suppressed IL-10 synthesis or compromised function. Mitogen-activated protein kinase (MAPK) cascades are crucial signaling pathways in the regulation of the host immune response to infection [36]. The up-regulation of map2K6 and down-regulation of mapk7 were evident at 3 h post *V. vulnificus* infection, which promotes an inflammatory response [37]. The same phenomenon was observed during infection of ivory shells with *V. harveyi* [35]. Similarly, several MAPK genes were significantly up-regulated at 3h, including *map3K2*, *map2K6*, *mapk13*, and *mapkapk3*; whereas *map2K7*, *mapk1*, and *mapk15* were down-regulated. The MAPK signaling pathway also regulates response to salinity stress [38,39], one of several signaling pathways responding to osmotic stress in the *Rachycentron canadum* [40] and *Lateolabrax maculatus* [41]. Additionally, MAPK14a has been associated with oxidative damage in *Ictalurus punctatus* under extreme salinity stress, highlighting the role of MAPK pathways in managing both osmotic and oxidative stress [42]. In contrast to the rapid role of JNK in molluscan stress responses [43,44,45], the down-regulated genes in *B. areolata* were primarily annotated to the JNK subfamily (e.g., MAPK1), participating in apoptosis or in cell proliferation. These expression patterns may suggest that this signaling module may restrain excessive inflammation via negative feedback regulation. According to our current results, we hypothesized that *map3K2* activation by bacterial infection leads to the phosphorylation of the downstream *map2K6*, and subsequent activating of *mapk13*, a molecule is involved in cellular stress responses (e.g., oxidative stress) and inflammation regulation. The MAP2K6-p38 MAPK might phosphorylate MAPKAPK3, which could potentially promote immune cell activation and the release of inflammatory cytokines (e.g., IL-1, TNF-α) to boost antimicrobial immunity stress responses. This proposed phosphorylation cascade is merely deduced from transcript profiles and requires further experimental validation.

In mammals, exposure to stress activates MAPK to regulate critical processes such as glycolysis, fatty acid oxidation, and energy metabolism, thereby maintaining cellular energy homeostasis [46]. Notably, the genes involved in carbon metabolism, lipid metabolism, and amino acid metabolism were widely down-regulated in *B. areolata* after infection. The activity of AMPK, depending on the down-regulation of *prkag2* and *prkaa2*, was restricted. Besides the down-regulation of *slc27a6* and *fabp3*, genes related to gluconeogenesis (*fbp*, *pepck*, and *creb*), transportation (*slc2a1/3/5*), and regulation of glycolysis (*pfk-2*) exhibited altered expression patterns, which may suggest suppressed gluconeogenesis and remodeled glycolytic flux. We speculate that these expression shifts may facilitate transport of glucose from hepatopancreatic cells toward immune effector cells, though this metabolic redistribution needs further verification. Meanwhile, down-regulation of *eef2k* and *lkb1* may relieve translational inhibition and weaken anabolic restriction, together accelerating the production of cytokines. These genes were down-regulated at 3 h and 24 h, indicating that the hepatopancreas selectively down-regulated basal metabolism genes that could be temporarily paused during the early immune response. Likewise, genes critical to energy supply and defense, such as *acnat2* and *grhpr*, were preferentially up-regulated in the early response. The liver is the primary organ for peroxisome fatty acid oxidation, where acyl-CoA N-Acyltransferase 2 (ACNAT2) is mainly involved in the amination reaction in peroxisomal lipid metabolism and plays a specific role in cellular lipid homeostasis [47]. Glyoxylate reductase/hydroxypyruvate reductase (GRHPR) is a key enzyme involved in glycolysis, glyoxylic acid metabolism, and detoxification [48]. Here, GLUT1 is encoded by *slc2a1*, a widely expressed glucose transporter, whose function is to mediate extracellular glucose transmembrane entry into cells, especially in the presence of high metabolic demand or stress [49]. In addition, OGT is a key enzyme that mediates the modification of protein O-linked β-N-acetylglucosamine, resulting in the modification of proteins such as inflammatory cytokines [50], HSP70 [51], and caspase-3 [52,53]. At 48 h, increased *slc2a1* (GLUT1) and *ogt* (O-GlcNAc transferase) were observed. These expression changes tentatively point to a potential coordinated host strategy to enhance nutrient uptake, rearrange metabolic distribution, and modulate immune signaling during the mid-phase of infection.

Autophagy is a conserved process that degrades damaged components (such as abnormal proteins, senescent organelles) or pathogens [54,55]. As part of the innate immune system, it is rapidly activated upon pathogen invasion in aquatic species [54]. In Crassostrea hongkongensis [56] and Crassostrea gigas [57], autophagy serves as a crucial innate immune response against Vibrio infection. Interestingly, although most related genes were generally down-regulated until 48 h, *tbk1*, *snap29*, and *rps27a* were specifically up-regulated at 3 h. Specifically, TANK binding kinase 1 (TBK1) is a key activator of selective autophagy (xenophagy), recognizing and targeting specific pathogens [58], whereas synaptosome-associated protein 29 (*snap 29*) can directly promote autophagosome–lysosome membrane fusion and the efficient degradation of bacteria [59,60]. Ribosome protein S27A is reported to mediate bacterial modification via ubiquitination to facilitate TBK1 targeting. Autophagy lysosomes contain more than 50 hydrolytic enzymes, such as proteases, lipases, and nucleases, that are critical for autophagic degradation [61]. They can digest and decompose foreign substances, senescent or damaged organelles, and misfolded proteins in cells, to maintain the stability of the intracellular environment. The lysosome pathway was identified as a significantly enriched key pathway in response to acute ammonia toxicity [13] and *V. harveyi* infection [35]. Similarly, in the current study, the lysosome pathway was one of the most significantly enriched pathways from 3 h to 48 h, only reverting to normal levels at 72 h. Additionally, related genes such as *clathrin* (CLTA/B) for endocytosis, lysosomal proteases (cathepsins), and other acid hydrolases were widely down-regulated. However, *psap* (encodes prosaposin) and *npc2* (Niemann–Pick disease type C) were dramatically up-regulated at 24 h and 48 h, respectively. Saposins assist with the degradation of pathogen lipids, while NPC2 is a key molecule in lysosomal cholesterol efflux, which prevents lipid overload toxicity in lysosomes [62]. Although these two genes target different lipid metabolism pathways (PSAP mediates sphingolipid degradation and NPC2 regulates cholesterol transport), up-regulation of their expression reflects the synergistic strategy of the host to enhance defense by remodeling the lysosomal lipid metabolism network [63]. This synergistic effect potentially reveals the critical role of lysosomes as lipid-immune hubs. Moreover, *ctsl* (encodes cathepsin L) was uniquely up-regulated at 72 h, possibly participating in the removal and reconstruction of damaged tissue as well as the regulation of inflammation regression. This may indicate that lysosomes, which are involved in stress response, endocytosis regulation, and protein and lipid degradation, act as functional effectors in the ivory shell’s response to *V. tubiashii* infection.

Inhibitors of apoptosis proteins (IAPs) are a family of homologous proteins with anti-apoptotic functions [64,65]. Members of the human IAP family include BIRC1-8, although the X-linked inhibitor of apoptosis protein (XIAP, BIRC4) is the most potent endogenous member of the family [66]. *Drosophila* inhibitor of apoptosis protein (DIAP) is homologous to XIAP in vertebrates and is mainly found in invertebrates such as drosophila [67]. Specifically, XIAP exerts its anti-apoptotic effects by directly binding to caspases (caspase-3, -7, and -9) and/or activating the nuclear factor kappa B (NF-κB) pathway, which promotes the release of cytokines (IL-1β and TNF-α) and enhances innate immunity [68]. Transcription factor AP-1 (JUN) and caspase 3 peaked at 3 h and were accompanied by the reduction of *bcl-2*, *birc 2/3/7*, and *diap2* until 24 h or 48 h. A short increase of *xiap* and *diap2* was observed at 3 h, followed by the continuous reduction of IAPs, substrates (PARP and Fodrin), and caspase 3/8 at 24 h and 48 h. This suggests that apoptosis was activated after *V. tubishii* infection, even with a compensatory increase of *xiap* during early infection that failed to prevent apoptotic initiation. The co-decline of APIs and caspases in the later stage of infection may indicate the end of the apoptotic process rather than the sustained inhibition of apoptosis.

Heat shock proteins (HSPs) are known to increase under different stressful conditions such as cold, osmotic, and oxidative stress, hypoxia, exposure to toxic substances, or infections [69,70]. As molecular chaperones, they protect proteins from denaturation and assist in the refolding or degradation of aberrant proteins, which are proposed to act as danger signals and immune-regulatory molecules [71]. Here, HSP70 and HSP90 are the most widely identified in mollusks under different stresses. Heat shock protein A5, also known as endoplasmic reticulum chaperone BiP (HSPA5), is a member of the heat shock protein 70 (HSP70) family and a core regulator of the endoplasmic reticulum stress response [72]. Protein disulfide isomerase A4 (Pdia4) is a member of the protein disulfide isomerase (PDI) family, also located in the endoplasmic reticulum (ER), and is involved in protein folding and regulation of redox homeostasis. The notable increase of *hspa5* and *pdia4* in early infection (3 h and/or 24 h) may enhance effective synthesis and secretion of immune-related proteins, prolong cell survival by inhibiting apoptotic signals, and promote the release of inflammatory cytokines through pathogen recognition. On the other hand, Hsp90 can be constitutive or inducible and is involved in protein maturation and degradation, signal transduction, and proteostasis under different stress conditions [73]. An increase in *hsp90a.1* was identified during early *V. tubiashii* infection in *B. areolata*, and together with the upregulation of E3 ubiquitin ligase. It is proposed that HSP90A.1 may target misfolded proteins and promote their degradation by collaborating with molecules of the ubiquitin-proteasome system or the autophagy pathway. Besides, genes related to the cytoskeleton, such as actin, were generally up-regulated during pathogen infection, suggesting that the cytoskeleton plays an essential role in the innate immunity of the ivory snail. In contrast, genes related to the cytochrome P450 (CYP450) family were generally down-regulated, indicating the total dysregulation of liver detoxification and metabolic functions (e.g., fatty acids, cholesterol).

Finally, the variation of inflammatory cytokines in *B. areolata* at the protein level was elucidated. Notably, expression of TNF-α, IL-1, and IL-8 was instantaneously induced by *V. tubiashii* infection (3 h), while IL-6 was induced afterward (24 h). Except for IL-8, TNF-α, IL-1, and IL-6 were increasingly expressed during the middle stage of infection (24 h and 48 h), indicating their function in the initiation and activation of inflammation. Mammalian IL-17 plays a pro-inflammatory role in the adaptive immune system and regulates innate immunity, increasing the expression of chemokines and anti-microbial molecules [74]. Molluscan IL-17 was reported to regulate the expression and release of humoral factors in *Biomphalaria glabrata* [75], *Crassostrea gigas* [76], *Mytilus coruscus* [77], and *Sepiella japonica* [78]. Importantly, IL-17 was elevated only at 72 h in *B. areolata*, which may imply its inhibition of an excessive immune response, regulation of immune balance, and role in the repair of inflammatory damage. It should be noted that the shellfish ELISA kits were only verified via pre-experimental functional tests rather than full antibody specificity identification, so the inference remains tentative.

## 5. Conclusions

This study describes the correlative changes of histopathology, transcriptome, enzyme activity, and cytokines in *B. areolata* infected with *V. tubiashii*. Critical time points after bacterial infection (3 h, 24 h, 48 h, and 72 h) were selected to show early, medium, and late biological host responses. Infection of *V. tubiashii* triggered host responses: acute immune activation and oxidative stress occurred at the early stage; persistent immune activity and progressive tissue damage were observed in the middle stage; the late stage presented stabilized transcription, inflammatory imbalance, and residual oxidative injury. A large set of DEGs and enriched immune pathways were identified. Multiple key functional genes from the *birc* and *hsp* families may participate in *B. areolata* immune response. The key genes and pathways obtained in this study are recommended as candidate molecules for future functional validation and mechanistic investigation. Enzymatic activity and inflammatory cytokines were induced during early infection, which may accelerate the initiation of host immune defense at the molecular level. However, whether such a molecular response improves disease outcome and survival needs further experimental verification. Nevertheless, these results provide important insight into the anti-bacterial responses of shellfish and the network of immune-related signaling pathways during bacterial infection. This provides preliminary clues for the molecular basis of immune mechanisms during *B. areolata* infection.

It is also important to acknowledge several experimental limitations of the present study. Sample pooling obscures individual variation, and our artificial infection system and detection tools cannot fully mimic natural infection scenarios. The commercial ELISA kits have only undergone partial cross-species validation for *B. areolata*, and full species-specific verification is still lacking. Histopathological evaluation was limited to qualitative observation. Despite these restrictions, the integrated multi-index results effectively illustrate the staged immune and metabolic responses of *B. areolata* to bacterial infection. Follow-up work will be improved.

## Figures and Tables

**Table 1:** Summary of sequencing results.

Sample	Raw Reads (bp)	Clean Reads (bp)	Q20 (%)	Q30 (%)	GC (%)	Mapped (%)
PBS-1	6,071,805,300	5,918,095,397	97.82	94.32	49.06	82.56
PBS-2	8,354,126,100	8,030,860,223	97.48	93.46	50.00	83.75
PBS-3	6,246,167,700	6,110,452,834	97.59	93.66	48.94	82.55
3 h-1	7,299,052,800	7,128,949,218	97.41	93.39	46.44	79.56
3 h-2	5,483,865,300	5,383,604,035	97.49	93.56	46.95	80.26
3 h-3	6,277,087,800	6,142,031,739	97.35	93.14	45.00	80.32
24 h-1	6,053,256,600	5,944,760,541	97.83	94.20	43.92	77.33
24 h-2	5,868,210,000	5,728,036,948	97.86	94.42	42.11	80.59
24 h-3	5,409,107,400	5,305,175,002	97.56	93.66	41.89	79.77
48 h-1	5,645,967,900	5,529,360,577	97.45	93.38	44.02	80.50
48 h-2	6,962,169,000	6,813,070,316	97.52	93.62	45.24	80.72
48 h-3	7,926,644,700	7,755,411,984	97.89	94.48	44.20	80.32
72 h-1	5,555,948,400	5,438,994,260	97.39	93.37	44.71	78.04
72 h-2	6,080,933,700	5,920,664,595	97.59	93.75	46.82	80.77
72 h-3	7,223,701,800	6,963,421,777	97.61	93.86	47.60	81.42

**Table 2:** Primer list for quantitative RT-PCR verification.

Gene Name	Forward Primer (5′-3′)	Reverse Primer (5′-3′)	Amplicon Length (bp)
*hpn*	GGCAGGCAGTTCCAGTCTATG	CAGTCAAGCCCTCTGTCCAA	159
*hsp90a.1*	TGTGGGTGATGTGATGTGGG	ATTCCTGCTGGTCCTCCTTC	146
*lysoz3*	TTTCTGACAATCGTTCGTCCTT	CTGGTCCGAAAGTGGCGTAT	179
*pgrp-sc2*	AGCCCTTTGTCTGCGGTAAT	CACTCCGTTTGGCACTCATC	159
*gm2a*	CTGCTGCCACTGCTTCTTCT	ATGCTGGTATAGCCGCGTAA	225
*hspa5*	TCCATAACCCACCGAACGC	CCTGCTAGTGCCTGAACCC	102
*heph*	TCGGGTCCACTCTGTTTACG	CAGGGAAGGGAGGCTATTTT	184
*vcan*	TAGCGCCTATGCTCGGTAGA	TTCGGTGCGTTATGGAAACA	218
*cd209e*	GTCGGTCGTCTTATGGTCGTA	GTCGGTTTGTGGTGGATTTG	261
*klh2*	GCCAATGACGAGACCTACGA	AATCCCGAATCCCACCTACA	217
*pnlip*	AGACCACGAGTTCGCAGCAT	CGCCGATAGAAAGTCATCCC	107
*i-2*	CAGTCTGATTTACGCTGGGATA	CATGCTCTGTGGGCTAGGTG	233
*lec-1*	TCACCTATCAGTTAGCGAGCAT	TAAGGGCCGAAACACTTGAC	286
*tnfsf10*	CGAACCTGTGCGGGAAGAT	CAGTGACGCCTCCTTGAGC	109
*fth1-a*	GAAGAGCGTCAACCAGTCCC	GACCGACCGACCTGCTAACT	115
*actin*	TTTCGCACCAGTCATTCACA	CTTCCTCTTTCGCTTCGTCA	155

**Table 3:** The top 20 significant immune-related pathways at different time points after infection.

Time	Pathways	Number of DEGs
3 h	NOD-like receptor signaling pathway	29
	T cell receptor signaling pathway	19
	Natural-killer-cell-mediated cytotoxicity	13
	Leukocyte transendothelial migration	19
	Rheumatoid arthritis	8
	Th1 and Th2 cell differentiation	10
	Platelet activation	22
	Th17 cell differentiation	9
	Toll-like receptor signaling pathway	15
	Inflammatory bowel disease	3
	IL-17 signaling pathway	10
	B cell receptor signaling pathway	11
	Fc epsilon RI signaling pathway	9
	Autoimmune thyroid disease	1
	C-type lectin receptor signaling pathway	15
	Primary immunodeficiency	2
	Toll and Imd signaling pathway	7
	Chemokine signaling pathway	16
	RIG-I-like receptor signaling pathway	6
	Fc gamma R-mediated phagocytosis	13
24 h	Th17 cell differentiation	21
	T cell receptor signaling pathway	36
	IL-17 signaling pathway	25
	Toll-like receptor signaling pathway	33
	B cell receptor signaling pathway	27
	RIG-I-like receptor signaling pathway	19
	NOD-like receptor signaling pathway	48
	Toll and Imd signaling pathway	19
	Intestinal immune network for IgA production	4
	Th1 and Th2 cell differentiation	17
	Natural-killer-cell-mediated cytotoxicity	21
	Leukocyte transendothelial migration	31
	Chemokine signaling pathway	36
	Cytosolic DNA-sensing pathway	17
	Rheumatoid arthritis	12
	Fc epsilon RI signaling pathway	18
	C-type lectin receptor signaling pathway	31
	Fc gamma R-mediated phagocytosis	29
	Complement and coagulation cascades	9
	Autoimmune thyroid disease	2
48 h	NOD-like receptor signaling pathway	31
	Th17 cell differentiation	12
	B cell receptor signaling pathway	15
	IL-17 signaling pathway	13
	Th1 and Th2 cell differentiation	11
	Natural-killer-cell-mediated cytotoxicity	13
	Toll-like receptor signaling pathway	17
	Inflammatory bowel disease	4
	Toll and Imd signaling pathway	10
	T cell receptor signaling pathway	16
	Intestinal immune network for IgA production	2
	Fc epsilon RI signaling pathway	10
	RIG-I-like receptor signaling pathway	8
	Complement and coagulation cascades	5
	Rheumatoid arthritis	6
	Chemokine signaling pathway	17
	Cytosolic DNA-sensing pathway	7
	C-type lectin receptor signaling pathway	13
	Antigen processing and presentation	4
	Leukocyte transendothelial migration	12
72 h	Rheumatoid arthritis	4
	IL-17 signaling pathway	5
	Th17 cell differentiation	3
	Toll and Imd signaling pathway	3
	B cell receptor signaling pathway	3
	Antigen processing and presentation	2
	Toll-like receptor signaling pathway	3
	Primary immunodeficiency	1
	T cell receptor signaling pathway	3
	Inflammatory bowel disease	1
	NOD-like receptor signaling pathway	4
	C-type lectin receptor signaling pathway	2
	Th1 and Th2 cell differentiation	1
	Natural-killer-cell-mediated cytotoxicity	1
	Systemic lupus erythematosus	1

Note: Pathways were ranked automatically by enrichment score without manual filtering. KEGG pathway nomenclature is based on vertebrate data. All functional interpretations are limited to conserved innate immune signaling components of mollusks.

**Table 4:** Key genes in most significant correlated modules analyzed by WGCNA.

Gene ID	Module	kTotal	kWithin	Symbol	Description
BABareV306129	turquoise	1846	1594	*adam12*	disintegrin and metalloproteinase domain-containing protein 12
BABareV310784	turquoise	1797	1563	*prrc2a*	PRRC2A
BABareV305867	turquoise	1806	1548	*rrbp1*	ribosome-binding protein 1
BABareV309951	turquoise	1817	1551	*clstn1*	calsyntenin-1
BABareV322707	turquoise	1759	1522	*aco1*	cytoplasmic aconitate hydratase
BABareV304105	turquoise	1795	1536	*igf2r*	cation-independent mannose-6-phosphate receptor
BABareV301913	turquoise	1741	1499	*add1*	Adducin 1
BABareV325031	turquoise	1724	1483	*bsg*	I-type lectin
BABareV303418	turquoise	1740	1511	*cdh23*	cadherin-23
BABareV301786	turquoise	1708	1476	*nrg*	neuroglian
BABareV319683	turquoise	1744	1493	*pat-3*	integrin beta 3
BABareV303009	turquoise	1704	1487	*reep5*	receptor expression-enhancing protein 5
BABareV317442	royal blue	199	57	*pzp*	alpha-2-macroglobulin
BABareV302540	royal blue	147	59	*csrp2*	cysteine and glycine-rich protein
BABareV303394	royal blue	177	54	*zip*	myosin heavy chain, non-muscle
BABareV304414	dark green	211	60	*acp7*	acid phosphatase 7
BABareV308816	dark green	224	48	*pim1*	serine/threonine-protein kinase pim-1
BABareV313062	dark green	164	58	*tbk1*	TANK-binding kinase 1

**Table 5:** Summary of top immune-related DEGs in PPI by node degree value.

Symbol	Gene Name	Function	Degree
*hsp90a.1*	Heat Shock Protein 90 Alpha Family Class A Member 1	Chaperones to assist in the folding and activation of client proteins	95
*mapk15*	Mitogen-Activated Protein Kinase 15	Cell proliferation, differentiation, apoptosis, and stress responses (such as oxidative stress, DNA damage)	92
*pik-1*	Phosphoinositide Kinase 1	Cytoskeletal reorganization, membrane trafficking, cell polarity establishment, and signal transduction	91
*rac1*	Ras-Related C3 Botulinum Toxin Substrate 1	Cytoskeletal reorganization of actin, cell migration, invasion and morphogenesis, phagocytosis, and immune cell activation	87
*ced-10*	Cell Death Abnormality 10	Small GTPases in Rho family, which are homologous genes of RAC1 in invertebrates, involving in Cell migration and phagocytosis	87
*Rhob*	Ras Homolog Gene Family Member B	Cytoskeleton and cell adhesion, cell cycle regulation, apoptosis, and angiogenesis	86
*btk*	Bruton Tyrosine Kinase	A key kinase in the B-cell receptor signaling pathway	84

## Data Availability

The raw sequencing data generated in this study have been deposited in the NCBI BioProject database under accession number PRJNA1468929 and are publicly available via the NCBI Sequence Read Archive (SRA).

## Abbreviations

The following abbreviations are used in this manuscript:

DEGs	differentially expressed genes
β-actin	beta-actin
SOD	superoxide dismutase
CAT	catalase
ACP	acid phosphatase
AKP	alkaline phosphatase
LZM	lysozyme
POD	peroxidase
GSH-PX	glutathione peroxidase
LPS	lipase
AMS	amylase
MDA	malondialdehyde
IL-1	interleukin 1
TNF-α	tumor necrosis factor alpha
WGCNA	weighted gene co-expression network analysis
PPI	protein–protein interaction
